# Crystal structure of *catena*-poly[[tri­methyl­tin(IV)]-μ-2-(2-nitro­phen­yl)acetato-κ^2^ *O*:*O*′]

**DOI:** 10.1107/S205698901500198X

**Published:** 2015-02-04

**Authors:** Muhammad Danish, Muhammad Nawaz Tahir, Sana Iftikhar, Muhammad Asam Raza, Muhammad Ashfaq

**Affiliations:** a Department of Chemistry, Institute of Natural Sciences, University of Gujrat, Gujrat 50700, Pakistan; b Department of Physics, University of Sargodha, Sargodha, Punjab, Pakistan

**Keywords:** crystal structure, one-dimensional coordination polymer, tri­methyl­tin(IV) complex, π–π inter­action

## Abstract

In the title one-dimensional coordination polymer, [Sn(CH_3_)_3_(C_8_H_6_NO_4_)]_*n*_, the Sn^IV^ atom is coordinated by three methyl C atoms and two carboxyl­ate O atoms (one symmetry generated), resulting in an almost regular SnC_3_O_2_ trigonal pyramid. The C atoms occupy the equatorial sites and the O atoms occupy the axial sites. In the ligand, the dihedral angles between the benzene ring and the pendant acetate and nitro groups are 57.7 (1) and 36.9 (3)°, respectively. The bridging ligand leads to [010] chains in the crystal, with adjacent metal atoms related by a 2_1_ screw axis. A weak π–π inter­action exists between the centroids of symmetry-related benzene rings at a distance of 3.9131 (19) Å.

## Related literature   

For related structures see: Tahir *et al.* (1997*a* ▸,*b* ▸); Tariq *et al.* (2013 ▸); Yang *et al.* (2009 ▸); Wen *et al.* (2009 ▸); Danish *et al.* (2015 ▸).
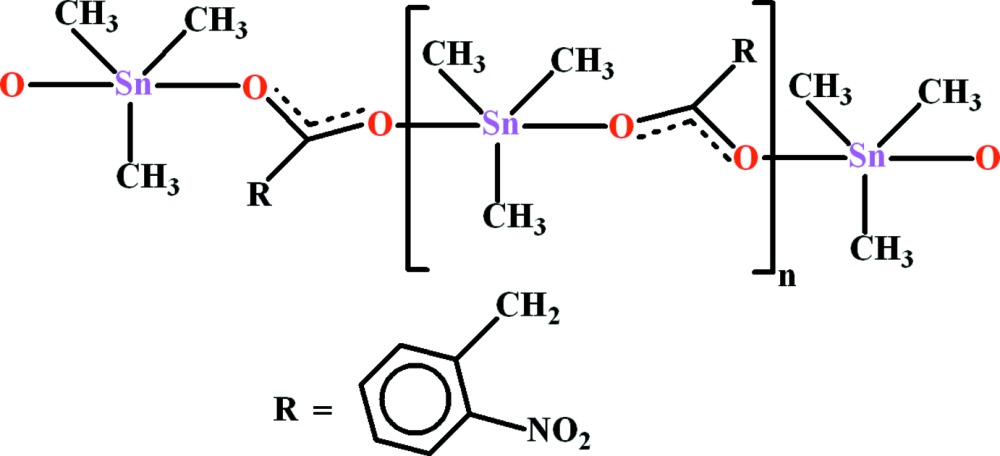


## Experimental   

### Crystal data  

[Sn(CH_3_)_3_(C_8_H_6_NO_4_)]
*M* *_r_* = 343.93
Monoclinic, 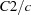

*a* = 12.6068 (5) Å
*b* = 9.9798 (4) Å
*c* = 22.7581 (9) Å
β = 100.174 (2)°
*V* = 2818.25 (19) Å^3^
*Z* = 8
Mo *K*α radiation
μ = 1.82 mm^−1^
*T* = 296 K
0.40 × 0.32 × 0.28 mm

### Data collection  

Bruker Kappa APEXII CCD diffractometer
Absorption correction: multi-scan *SADABS* (Bruker, 2005 ▸) *T*_min_ = 0.532, *T*_max_ = 0.631
11546 measured reflections
3074 independent reflections
2734 reflections with *I* > 2σ(*I*)
*R*_int_ = 0.022

### Refinement  

*R*[*F* ^2^ > 2σ(*F* ^2^)] = 0.025
*wR*(*F* ^2^) = 0.057
*S* = 1.10
3070 reflections
157 parameters
H-atom parameters constrained
Δρ_max_ = 0.74 e Å^−3^
Δρ_min_ = −0.53 e Å^−3^

### 

Data collection: *APEX2* (Bruker, 2007 ▸); cell refinement: *SAINT* (Bruker, 2007 ▸); data reduction: *SAINT*; program(s) used to solve structure: *SHELXS97* (Sheldrick, 2008 ▸); program(s) used to refine structure: *SHELXL97* (Sheldrick, 2008 ▸); molecular graphics: *ORTEP-3 for Windows* (Farrugia, 2012 ▸) and *PLATON* (Spek, 2009 ▸); software used to prepare material for publication: *WinGX* (Farrugia, 2012 ▸) and *PLATON* (Spek, 2009 ▸).

## Supplementary Material

Crystal structure: contains datablock(s) global, I. DOI: 10.1107/S205698901500198X/hb7359sup1.cif

Structure factors: contains datablock(s) I. DOI: 10.1107/S205698901500198X/hb7359Isup2.hkl

**Fig. 1.**
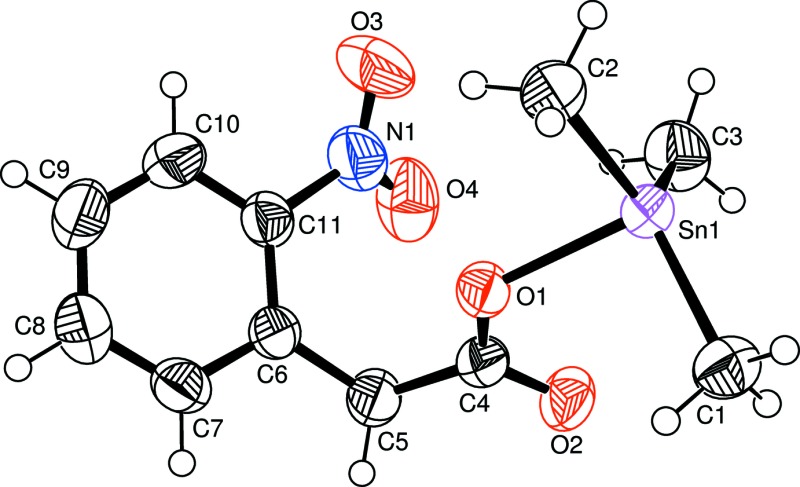
View of the title compound with displacement ellipsoids drawn at the 50% probability level.

. DOI: 10.1107/S205698901500198X/hb7359fig1.tif
View of the title compound with displacement ellipsoids drawn at the 50% probability level.

**Fig. 2.**
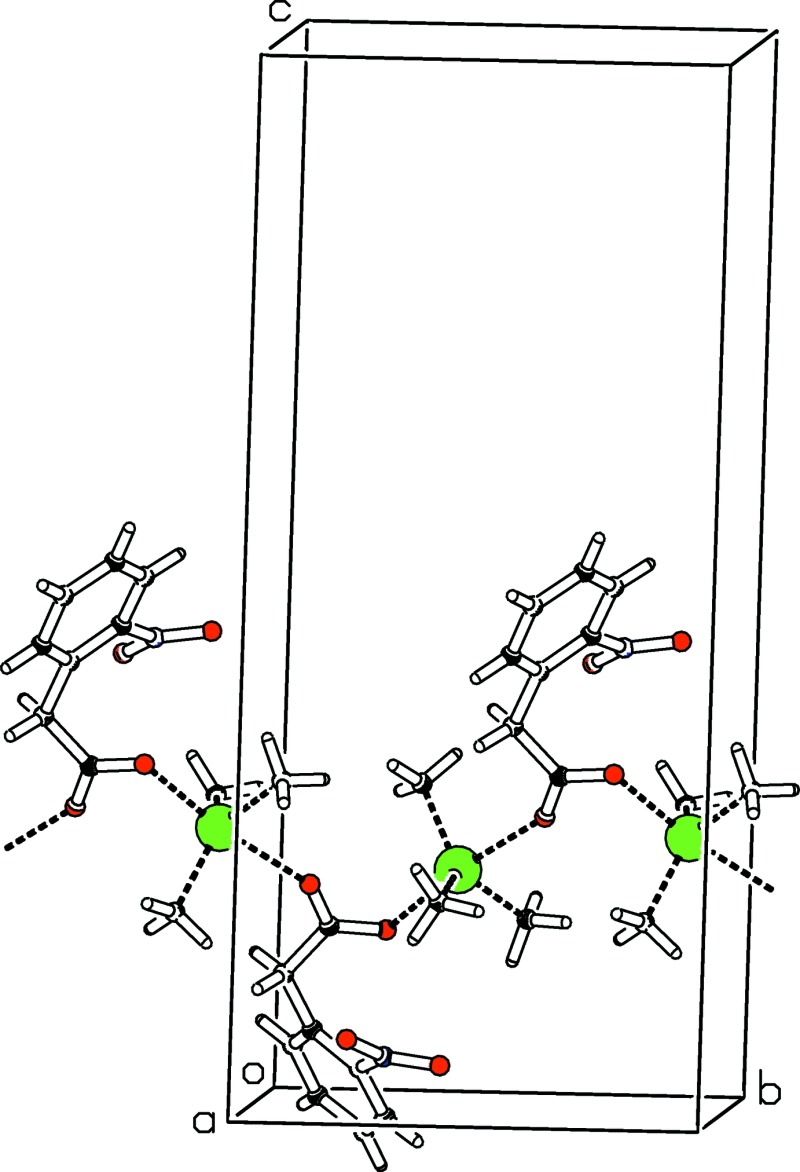
Fragment of an [010] chain in the structure of the title compound.

. DOI: 10.1107/S205698901500198X/hb7359fig2.tif
Fragment of an [010] chain in the structure of the title compound.

CCDC reference: 1046314

Additional supporting information:  crystallographic information; 3D view; checkCIF report

## Figures and Tables

**Table 1:** Selected bond lengths ()

Sn1C3	2.114(3)
Sn1C2	2.120(3)
Sn1C1	2.121(3)
Sn1O1	2.1970(18)
Sn1O2^i^	2.359(2)

Symmetry code: (i) 
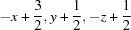
.

## supplementary crystallographic information

### S1. Comment

The tin complex (I), (Fig. 1) is in continuation of synthesizing various metal complexes with (2-nitrophenyl)acetic acid. In this context, we have reported the cobalt complex namely "Tetraaquabis((2-nitrophenyl)acetato-O) cobalt(II)" (Danish *et al.*, 2015).

The crystal structures of *catena*-Poly[[trimethyltin(IV)]-µ-2-(2-chlorophenyl) acetato] (Wen *et al.*, 2009), *catena*-[bis(µ2–3-(2- fluorophenyl)-2-methylprop-2-enoato)-hexamethyl-di-tin] (Tariq *et al.*, 2013), *catena*-poly[[trimethyltin(IV)]-µ-2-(3-thienyl)acetato] (Yang *et al.*, 2009), *catena*-((µ2–2-(3-benzoylphenyl)propanoato-)- trimethyl-tin(iv)) (Tahir *et al.*, 1997*a*), {2-[(2,3-Dimethylphenyl)amino] benzoato-*O*:*O*'}trimethyltin(IV) (Tahir *et al.*, 1997*b*) have been published which are related to the title compound due to coordination around the tin.

The Sn atom has a distorted trigonal bipyramidal geometry. The basal plane consists of three methyl groups and the apical position are occupied by the O-atoms of two carboxylate ligands. The Sn atom is 0.1082 (20) Å out of the equatorial plane towards the more strongly bound O1 atom. The Sn—O bond lengths are significantly different [Snl–O1 2.197 (2) and Snl–O2 2.359 (2) Å]. In the asymmetric unit the acetato moiety *A* (O1/C4/C5/O2), benzene ring *B* (C6–C11) are planar with r.m.s. deviation of 0.0020 and 0.0059 Å, respectively. The dihedral angle between A/B is 57.727 (115)°. The nitro group is oriented at a dihedtal angle of 36.896 (298)° with the benzene ring. The molecules form one-dimensional polymeric chains (Fig. 2) running along the crystallographic *b*-axis. There exist a π–π interaction between *Cg*1···*Cg*1^i^ [i = 1 - *x*, -*y*, -*z*] at a distance of 3.9131 (19) Å, where *Cg*1 is the centroid of benzene ring.

### S2. Experimental

The silver salt (1.44 g, 0.01 *M*) of 2-nitrophenyl acetic acid was suspended in 50 ml chloroform in a round bottom flask equipped with condenser and magnetic stirrer. Trimethyltin chloride (0.995 g, 0.01 *M*) in 5 ml of chloroform was added under inert atmosphere and reflux the reaction mixture for 4 h. The reaction mixture was allowed to cool to room temperature and stayed overnight then filtered. The residue was washed with chloroform and collected. It was concentrated on rotary evaporated and kept for crystallization. Colourless prisms were obtained after a week.

### S3. Refinement

The H atoms were positioned geometrically (C—H = 0.93—0.97 Å) and refined as riding with *U*_iso_(H) = *xU*_eq_(C), where *x* = 1.5 for methyl and *x* = 1.2 for other H-atoms.

### Crystal data

**Table:**

[Sn(CH_3_)_3_(C_8_H_6_NO_4_)]	*F*(000) = 1360
*M**_r_* = 343.93	*D*_x_ = 1.621 Mg m^−^^3^
Monoclinic, *C*2/*c*	Mo *K*α radiation, λ = 0.71073 Å
*a* = 12.6068 (5) Å	Cell parameters from 2734 reflections
*b* = 9.9798 (4) Å	θ = 1.8–27.0°
*c* = 22.7581 (9) Å	µ = 1.82 mm^−^^1^
β = 100.174 (2)°	*T* = 296 K
*V* = 2818.25 (19) Å^3^	Prism, white
*Z* = 8	0.40 × 0.32 × 0.28 mm

### Data collection

**Table:**

Bruker Kappa APEXII CCD diffractometer	3074 independent reflections
Radiation source: fine-focus sealed tube	2734 reflections with *I* > 2σ(*I*)
Graphite monochromator	*R*_int_ = 0.022
Detector resolution: 7.80 pixels mm^-1^	θ_max_ = 27.0°, θ_min_ = 1.8°
ω scans	*h* = −16→15
Absorption correction: multi-scan *SADABS* (Bruker, 2005)	*k* = −12→12
*T*_min_ = 0.532, *T*_max_ = 0.631	*l* = −29→29
11546 measured reflections	

### Refinement

**Table:**

Refinement on *F*^2^	Primary atom site location: structure-invariant direct methods
Least-squares matrix: full	Secondary atom site location: difference Fourier map
*R*[*F*^2^ > 2σ(*F*^2^)] = 0.025	Hydrogen site location: inferred from neighbouring sites
*wR*(*F*^2^) = 0.057	H-atom parameters constrained
*S* = 1.10	*w* = 1/[σ^2^(*F*_o_^2^) + (0.0174*P*)^2^ + 4.9157*P*] where *P* = (*F*_o_^2^ + 2*F*_c_^2^)/3
3070 reflections	(Δ/σ)_max_ = 0.002
157 parameters	Δρ_max_ = 0.74 e Å^−^^3^
0 restraints	Δρ_min_ = −0.53 e Å^−^^3^

### Special details

**Table:**

Geometry. All e.s.d.'s (except the e.s.d. in the dihedral angle between two l.s. planes) are estimated using the full covariance matrix. The cell e.s.d.'s are taken into account individually in the estimation of e.s.d.'s in distances, angles and torsion angles; correlations between e.s.d.'s in cell parameters are only used when they are defined by crystal symmetry. An approximate (isotropic) treatment of cell e.s.d.'s is used for estimating e.s.d.'s involving l.s. planes.
Refinement. Refinement of *F*^2^ against ALL reflections. The weighted *R*-factor *wR* and goodness of fit *S* are based on *F*^2^, conventional *R*-factors *R* are based on *F*, with *F* set to zero for negative *F*^2^. The threshold expression of *F*^2^ > σ(*F*^2^) is used only for calculating *R*-factors(gt) *etc*. and is not relevant to the choice of reflections for refinement. *R*-factors based on *F*^2^ are statistically about twice as large as those based on *F*, and *R*-factors based on ALL data will be even larger.

### Fractional atomic coordinates and isotropic or equivalent isotropic displacement parameters (Å^2^)

**Table:**

	*x*	*y*	*z*	*U*_iso_*/*U*_eq_	
Sn1	0.69602 (2)	0.44371 (2)	0.23090 (2)	0.04257 (7)	
O1	0.61758 (15)	0.28730 (18)	0.17044 (8)	0.0471 (4)	
O2	0.72746 (19)	0.1326 (2)	0.21529 (9)	0.0624 (6)	
O3	0.6779 (3)	0.4159 (3)	0.04647 (14)	0.1042 (10)	
O4	0.7472 (2)	0.2230 (3)	0.07130 (12)	0.0876 (8)	
N1	0.6705 (3)	0.2954 (3)	0.05335 (12)	0.0681 (8)	
C1	0.6587 (3)	0.3576 (3)	0.30997 (13)	0.0573 (7)	
H1A	0.5941	0.3052	0.3003	0.086*	
H1B	0.6480	0.4275	0.3373	0.086*	
H1C	0.7170	0.3011	0.3281	0.086*	
C2	0.5890 (3)	0.5844 (3)	0.18216 (15)	0.0633 (8)	
H2A	0.5272	0.5954	0.2010	0.095*	
H2B	0.5664	0.5527	0.1421	0.095*	
H2C	0.6250	0.6690	0.1813	0.095*	
C3	0.8483 (3)	0.4203 (4)	0.20539 (16)	0.0690 (9)	
H3A	0.8964	0.3757	0.2367	0.104*	
H3B	0.8770	0.5068	0.1984	0.104*	
H3C	0.8406	0.3679	0.1695	0.104*	
C4	0.6539 (2)	0.1693 (3)	0.17451 (12)	0.0464 (6)	
C5	0.6056 (3)	0.0706 (3)	0.12693 (13)	0.0569 (8)	
H5A	0.6640	0.0225	0.1137	0.068*	
H5B	0.5639	0.0056	0.1450	0.068*	
C6	0.5342 (2)	0.1286 (3)	0.07277 (11)	0.0444 (6)	
C7	0.4315 (3)	0.0782 (3)	0.05457 (13)	0.0535 (7)	
H7	0.4080	0.0084	0.0761	0.064*	
C8	0.3627 (3)	0.1280 (4)	0.00569 (14)	0.0617 (8)	
H8	0.2946	0.0907	−0.0056	0.074*	
C9	0.3944 (3)	0.2319 (4)	−0.02628 (15)	0.0684 (9)	
H9	0.3480	0.2656	−0.0593	0.082*	
C10	0.4947 (3)	0.2861 (3)	−0.00945 (14)	0.0641 (8)	
H10	0.5163	0.3580	−0.0305	0.077*	
C11	0.5636 (2)	0.2338 (3)	0.03875 (12)	0.0490 (6)	

### Atomic displacement parameters (Å^2^)

**Table:**

	*U*^11^	*U*^22^	*U*^33^	*U*^12^	*U*^13^	*U*^23^
Sn1	0.04503 (11)	0.04110 (11)	0.04012 (11)	−0.00067 (8)	0.00347 (7)	0.00225 (8)
O1	0.0541 (11)	0.0384 (10)	0.0459 (10)	0.0023 (8)	0.0006 (8)	−0.0024 (8)
O2	0.0797 (15)	0.0481 (12)	0.0518 (12)	0.0101 (11)	−0.0096 (11)	0.0010 (9)
O3	0.116 (2)	0.093 (2)	0.104 (2)	−0.0586 (19)	0.0209 (18)	0.0090 (17)
O4	0.0527 (14)	0.133 (3)	0.0780 (17)	−0.0086 (16)	0.0135 (13)	−0.0147 (17)
N1	0.0668 (19)	0.086 (2)	0.0540 (16)	−0.0253 (17)	0.0186 (14)	−0.0068 (15)
C1	0.0634 (19)	0.0589 (18)	0.0512 (17)	0.0007 (15)	0.0141 (14)	0.0050 (14)
C2	0.066 (2)	0.0493 (17)	0.068 (2)	0.0012 (15)	−0.0085 (16)	0.0092 (15)
C3	0.0542 (19)	0.085 (2)	0.070 (2)	−0.0089 (17)	0.0177 (16)	−0.0059 (18)
C4	0.0551 (16)	0.0409 (14)	0.0416 (14)	−0.0017 (12)	0.0039 (12)	0.0019 (11)
C5	0.073 (2)	0.0393 (15)	0.0527 (17)	0.0006 (14)	−0.0041 (14)	−0.0015 (12)
C6	0.0546 (16)	0.0384 (13)	0.0396 (13)	−0.0023 (12)	0.0066 (12)	−0.0056 (11)
C7	0.0619 (18)	0.0480 (16)	0.0519 (16)	−0.0125 (13)	0.0139 (14)	−0.0047 (13)
C8	0.0505 (17)	0.074 (2)	0.0588 (19)	−0.0074 (16)	0.0050 (14)	−0.0105 (17)
C9	0.071 (2)	0.076 (2)	0.0523 (18)	0.0025 (18)	−0.0070 (16)	0.0032 (17)
C10	0.084 (2)	0.0593 (19)	0.0479 (17)	−0.0088 (18)	0.0076 (16)	0.0102 (15)
C11	0.0508 (16)	0.0533 (16)	0.0429 (14)	−0.0090 (13)	0.0088 (12)	−0.0046 (12)

### Geometric parameters (Å, º)

**Table:**

Sn1—C3	2.114 (3)	C3—H3A	0.9600
Sn1—C2	2.120 (3)	C3—H3B	0.9600
Sn1—C1	2.121 (3)	C3—H3C	0.9600
Sn1—O1	2.1970 (18)	C4—C5	1.510 (4)
Sn1—O2^i^	2.359 (2)	C5—C6	1.507 (4)
O1—C4	1.261 (3)	C5—H5A	0.9700
O2—C4	1.245 (3)	C5—H5B	0.9700
O2—Sn1^ii^	2.359 (2)	C6—C7	1.383 (4)
O3—N1	1.219 (4)	C6—C11	1.393 (4)
O4—N1	1.218 (4)	C7—C8	1.376 (4)
N1—C11	1.465 (4)	C7—H7	0.9300
C1—H1A	0.9600	C8—C9	1.367 (5)
C1—H1B	0.9600	C8—H8	0.9300
C1—H1C	0.9600	C9—C10	1.366 (5)
C2—H2A	0.9600	C9—H9	0.9300
C2—H2B	0.9600	C10—C11	1.375 (4)
C2—H2C	0.9600	C10—H10	0.9300
			
C3—Sn1—C2	117.01 (14)	Sn1—C3—H3C	109.5
C3—Sn1—C1	122.46 (14)	H3A—C3—H3C	109.5
C2—Sn1—C1	119.75 (13)	H3B—C3—H3C	109.5
C3—Sn1—O1	94.58 (11)	O2—C4—O1	122.8 (3)
C2—Sn1—O1	88.20 (10)	O2—C4—C5	119.7 (3)
C1—Sn1—O1	95.78 (10)	O1—C4—C5	117.5 (2)
C3—Sn1—O2^i^	85.57 (12)	C6—C5—C4	116.3 (2)
C2—Sn1—O2^i^	84.62 (10)	C6—C5—H5A	108.2
C1—Sn1—O2^i^	90.86 (10)	C4—C5—H5A	108.2
O1—Sn1—O2^i^	172.00 (7)	C6—C5—H5B	108.2
C4—O1—Sn1	120.00 (17)	C4—C5—H5B	108.2
C4—O2—Sn1^ii^	143.50 (19)	H5A—C5—H5B	107.4
O4—N1—O3	123.7 (3)	C7—C6—C11	115.7 (3)
O4—N1—C11	118.1 (3)	C7—C6—C5	119.8 (3)
O3—N1—C11	118.2 (3)	C11—C6—C5	124.4 (3)
Sn1—C1—H1A	109.5	C8—C7—C6	122.3 (3)
Sn1—C1—H1B	109.5	C8—C7—H7	118.9
H1A—C1—H1B	109.5	C6—C7—H7	118.9
Sn1—C1—H1C	109.5	C9—C8—C7	120.2 (3)
H1A—C1—H1C	109.5	C9—C8—H8	119.9
H1B—C1—H1C	109.5	C7—C8—H8	119.9
Sn1—C2—H2A	109.5	C10—C9—C8	119.6 (3)
Sn1—C2—H2B	109.5	C10—C9—H9	120.2
H2A—C2—H2B	109.5	C8—C9—H9	120.2
Sn1—C2—H2C	109.5	C9—C10—C11	119.7 (3)
H2A—C2—H2C	109.5	C9—C10—H10	120.1
H2B—C2—H2C	109.5	C11—C10—H10	120.1
Sn1—C3—H3A	109.5	C10—C11—C6	122.5 (3)
Sn1—C3—H3B	109.5	C10—C11—N1	116.6 (3)
H3A—C3—H3B	109.5	C6—C11—N1	120.9 (3)
			
Sn1^ii^—O2—C4—O1	156.5 (2)	C8—C9—C10—C11	1.3 (6)
Sn1^ii^—O2—C4—C5	−24.2 (5)	C9—C10—C11—C6	−1.8 (5)
Sn1—O1—C4—O2	6.9 (4)	C9—C10—C11—N1	178.8 (3)
Sn1—O1—C4—C5	−172.5 (2)	C7—C6—C11—C10	0.8 (4)
O2—C4—C5—C6	−167.8 (3)	C5—C6—C11—C10	−178.2 (3)
O1—C4—C5—C6	11.6 (4)	C7—C6—C11—N1	−179.7 (3)
C4—C5—C6—C7	−127.1 (3)	C5—C6—C11—N1	1.2 (4)
C4—C5—C6—C11	51.9 (4)	O4—N1—C11—C10	−143.4 (3)
C11—C6—C7—C8	0.5 (4)	O3—N1—C11—C10	36.1 (4)
C5—C6—C7—C8	179.6 (3)	O4—N1—C11—C6	37.2 (4)
C6—C7—C8—C9	−1.0 (5)	O3—N1—C11—C6	−143.3 (3)
C7—C8—C9—C10	0.0 (5)		

Symmetry codes: (i) −*x*+3/2, *y*+1/2, −*z*+1/2; (ii) −*x*+3/2, *y*−1/2, −*z*+1/2.
