# Supplementary figures and images for: Crystal structure of catena-poly[[tri­methyl­tin(IV)]-μ-2-(2-nitro­phen­yl)acetato-κ2 O:O′]
Source: Acta Crystallogr E Crystallogr Commun. 2015 Feb 4;71(Pt 3):m52–3. doi: 10.1107/S205698901500198X (PMC4350728; doi:10.1107/S205698901500198X)

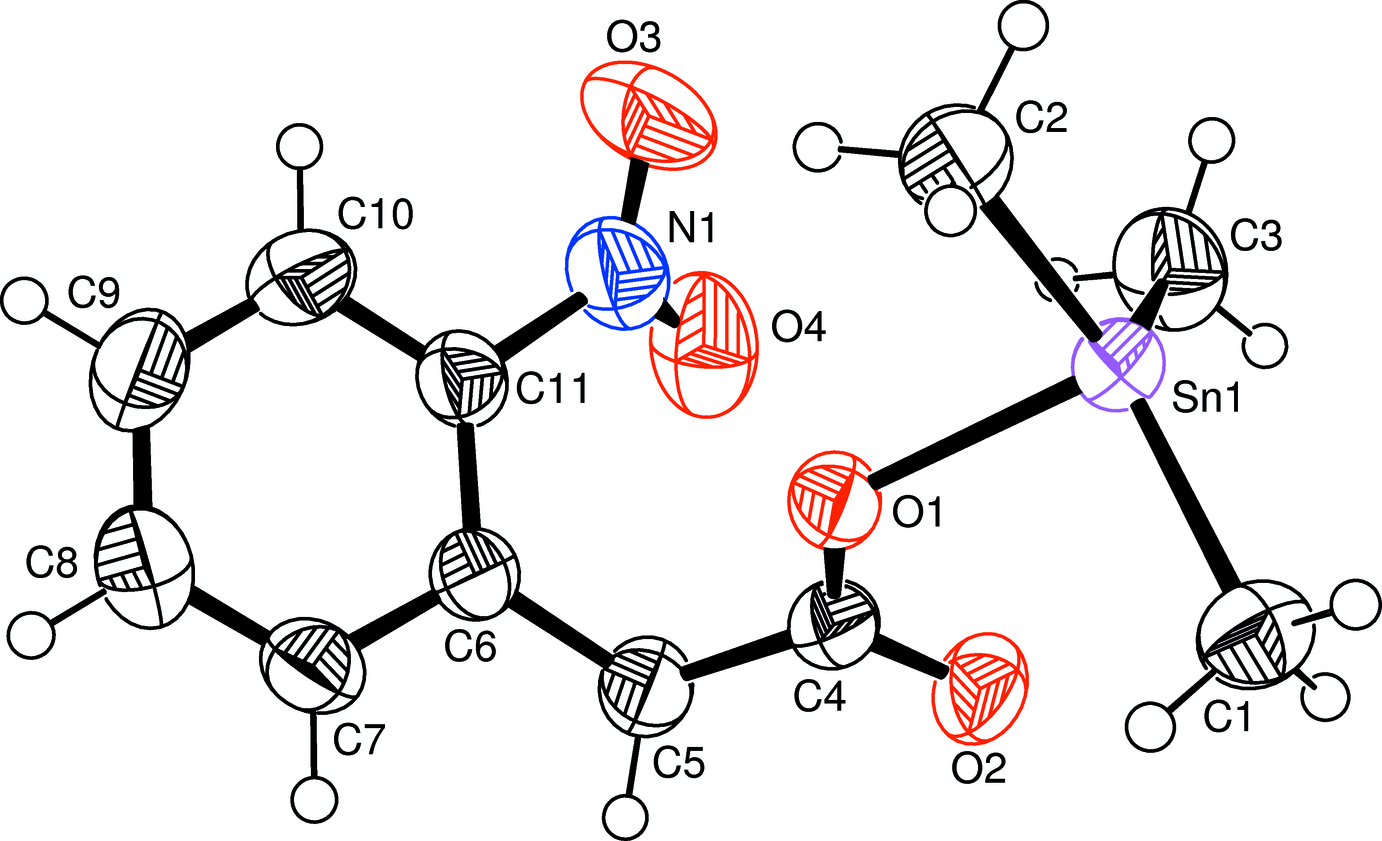

Supplement: Supplementary file 3 [file e-71-00m52-fig1.tif]

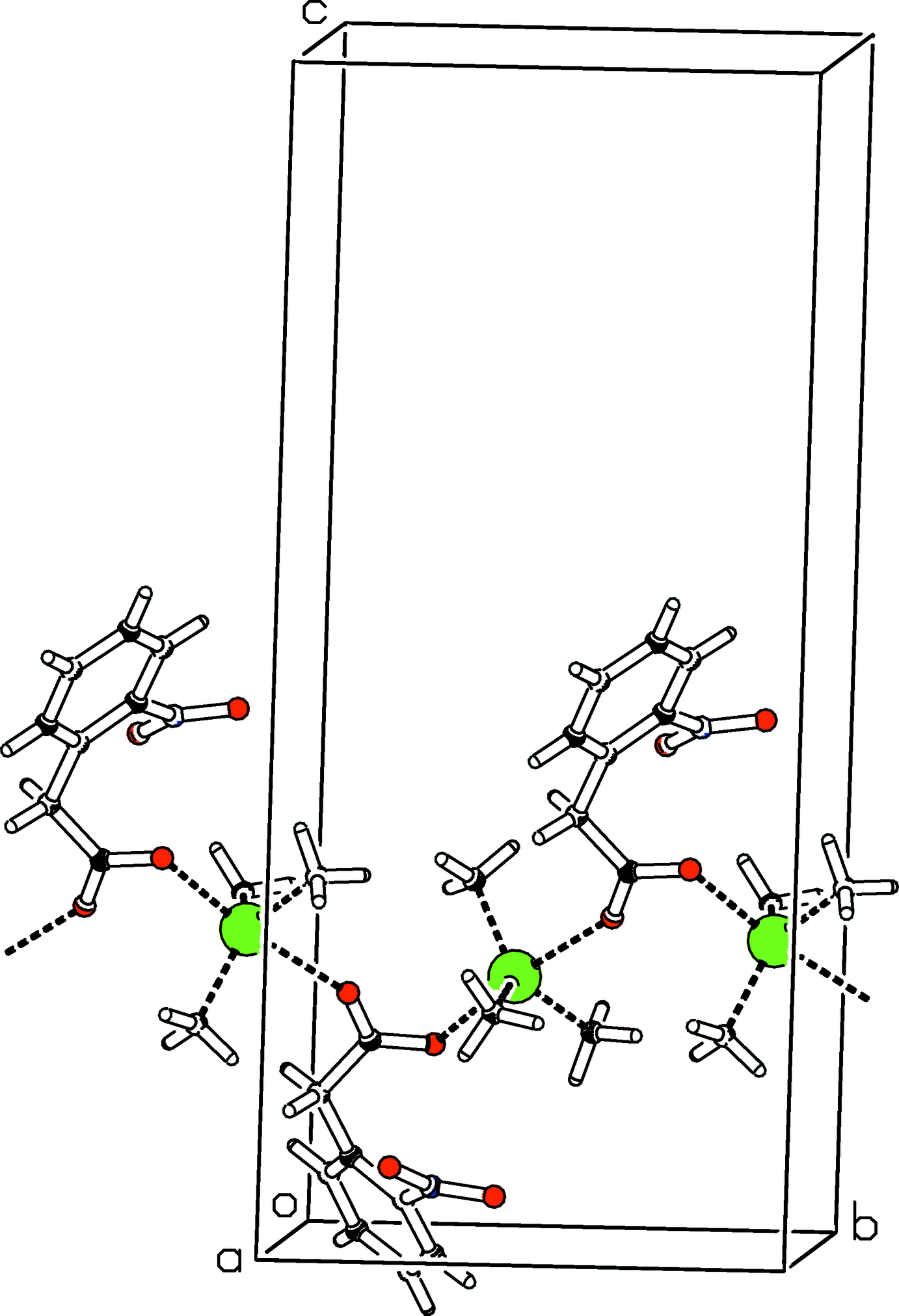

Supplement: Supplementary file 4 [file e-71-00m52-fig2.tif]
